# Strength Characteristics and Microstructure of Cement Stabilized Soft Soil Admixed with Silica Fume

**DOI:** 10.3390/ma14081929

**Published:** 2021-04-12

**Authors:** Nan Jiang, Changming Wang, Zeping Wang, Bailong Li, Yi-ao Liu

**Affiliations:** 1 College of Construction Engineering, Jilin University, Changchun 130026, China; jiangnan17@mails.jlu.edu.cn (N.J.); lbl19@mails.jlu.edu.cn (B.L.); liuya18@mails.jlu.edu.cn (Y.-a.L.); 2 Changchun Municipal Engineering Design & Research Institute, Changchun 130033, China; wangzp17@mails.jlu.edu.cn

**Keywords:** silica fume, cement stabilized soft soil, unconfined compressive strength, microstructure, pore distribution

## Abstract

Soft soil improvement is an important subject in civil engineering, and searching for an effective admixture is an important research. Silica fume (SF) is a kind of recycled material, it can be used in engineering as a pozzolanic material. The main objective of this study is to assess the effectiveness of industrial waste silica fume (SF) as an admixture to improve the cement stabilized soft soil. The unconfined compressive test (UCT) and scanning electron microscopy (SEM) test of cement stabilized soil with different SF contents and different curing times have been carried out. UCT after 28 days revealed that the addition of SF can effectively increase the strength of cement stabilized soil and reduce the amount of cement, and 1.5% SF content is considered optimum, excessive SF will not further increase the strength. SF helped to accelerate the cement hydration reaction and significantly improve the early-age strength of stabilized soil even at 3 days, which can improve construction efficiency in actual projects. SEM analyses shows that the proper SF content could make the hydration product calcium silicate hydrate gel (CSH) fill the pores and increase the strength of the material, but excessive SF will increase the large pores content of the material and reduce the strength. This provided a basis for application of SF in improving soft soil.

## 1. Introduction

Silica fume (SF) is an extremely fine dust produced in the production of silicon steel, ferrosilicon and other silicon-containing ores [1]. SF has a high content of SiO_2_ and a large specific surface area. Silica fume seriously damages the health of human lungs, its recycling and utilization are very necessary for environmental protection and human health. The composition of SF is mainly SiO_2_ and it is a highly active pozzolanic substance. Soft soil is widely distributed around coastlines and coastal areas. Due to its poor engineering characteristics such as large settlement, low bearing capacity, and poor stability, soft soil cannot be directly used in engineering. The improvement of soft soil is a severe challenge. The traditional improvement method of marine soft soil is the blow filling method, which has the disadvantage of long construction period. In addition, electroosmosis is also a method of stabilizing soft soil [2,3]. The deep mixing method is the most commonly used foundation improvement technology for soft soil, it has been applied in many countries, especially in Europe and North America [4,5]. Ordinary Portland cement (OPC) has high compressive strength, high bearing capacity and low compressibility. It is a commonly used binder for stabilizing soft soil in soft soil foundation improvement or dredging and filling land reclamation, which can improve the rigidity and strength of soft soil. A “cement-soil” pile will be formed to increase the strength after the cement is hydrated and hardened [6,7,8]. However, the use of cement as a stabilizer will have an adverse effect on the environment. One effect is that the manufacturing of cement causes 5–7% of global carbon dioxide emissions [9,10]. Another is that cemented soil usually appears as highly alkaline, disserving the ground water quality and vegetation [11,12]. Searching for an effective and inexpensive admixture is an important research in soil reinforcement [11]. Pozzolanic materials are SiO_2_ and Al_2_O_3_ based materials that can reduce the OPC required [13]. At present, fly ash, sludge and bottom ash have been used as pozzolanic materials to improve properties of soft soils [14,15]. The necessity of recycling and high SiO_2_ content make silica fume a good choice for reducing cement consumption. Because of the low density and strong adhesion, SF has been widely used in the reinforcement of concrete and cement mortar [16,17,18]. The increase of the compressive strength and tensile strength of concrete is the most famous function of SF, the elastic modulus and flexural strength of cement mortar are also improved [19,20]. The DSP (Densified Systems containing homogeneously arranged ultra-fine Particles) material developed by SF has a compressive strength of 100 MPa at 28 days, which is 3–5 times the strength of conventional concrete [21]. Pozzolanic materials react with calcium hydroxide (CH) to form a calcium silicate hydrate gel (CSH), and CSH provides a less corrosive environment, which helps to improve the durability of composite materials and makes it dense [22,23,24,25]. SF has been used as a pozzolanic material instead of cement to produce foamed concrete, which can compensate for the negative impact of vermiculite on mechanical values [26]. It is found that SF fills the pores and makes the structure dense during the hardening stage, when it is used to improve the performance of foam concrete or cement-based composites [27,28,29]. However, few studies on the use of SF as an admixture for cement stabilized soft soil have been reported. Studies have shown that fine particles can help increase early compressive strength due to their large specific surface area, the improvement of early compressive strength is a very important aspect in engineering construction, which is conducive to improving building quality and construction efficiency, reducing the risk of early cracks, and the building can be put into use earlier, therefore, SF particles can be considered to improve the early strength of materials [30].

In this study, silica fume was selected as the admixture of cement stabilized soil, and unconfined compression tests were carried out to study the process and mechanism of how to reduce the amount of cement and strengthen the compressive strength of cement soil. In addition to setting the curing age of 28 and 90 days, we also set samples of 3, 7 and 14 days. The microstructure characteristics of cement stabilized soils of different curing ages were observed, the process and mechanism of the cement soil reinforcement by silica fume content were analyzed, combined with the characteristics of pore distribution, the emergence of the optimal amount of silica fume incorporated was explained.

## 2. Materials and Methods

### 2.1. Materials

**Figure 1:**
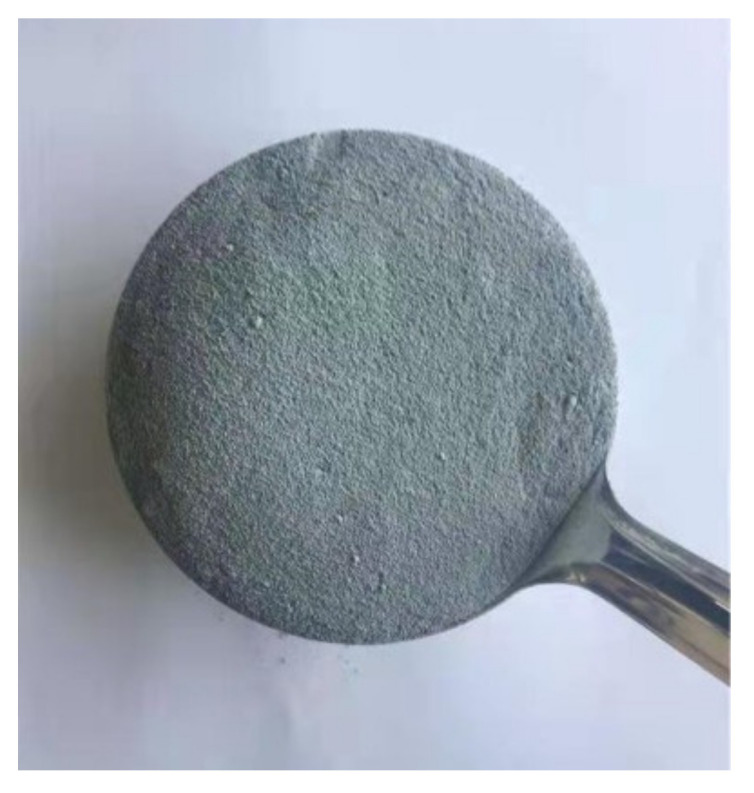
Silica fume sample particles.

The soft soil was taken from Zhejiang Province, China. The soil samples were collected at a depth of 5–8 m from the ground. Table 1 shows the basic physical properties of soft soil. The specific gravity and wet density of soil are 2.68 and 1.75 g/cm^3^, and the natural moisture content is 45.0%, which is higher than its liquid limit. Based on the Unified Soil Classification System, the soil is classified as inorganic clay with medium plasticity (CI). As shown in Figure 1, SF is off-white particles with the grain size of 0.1–0.2 μm, supplied by Borun Casting Material Ltd. (Henan, China), has a specific surface area of 25,000 m^2^/kg. Type I OPC is produced by Yatai Group (Changchun, China) with the specific surface area of 360 m^2^/kg, meeting the ASTM C150 [31].

Table 2 shows the chemical composition of soft soil, SF and OPC, which are obtained through X-ray fluorescence (XRF) spectrometry (PANalytical, Almelo, The Netherlands). The total SiO_2_ content of soft soil exceeds 60%, followed by 18.24% Al_2_O_3_ and 8.09% Fe_2_O_3_. All compositions of soft soil are considered inert relative to those of SF and OPC. The SF contains 95.02% SiO_2_, with minor amounts of Al_2_O_3_ and Fe_2_O_3_, which is higher than the limited value specified by ASTM C618 for a class N pozzolan material [32]. Active oxides such as SiO_2_ and Al_2_O_3_ play a very important role in the pozzolanic reaction of cement stabilized soil, thus, the SF had a high pozzolanic composition and could be used to reduce the amount of OPC and improve the quality of soft soil. Based on chemical composition, OPC mainly consists of CaO (64.29%), with minor amounts of SiO_2_ (21.75%), Al_2_O_3_ (5.66%) and Fe_2_O_3_ (4.63%) oxides. In addition, chemical analysis indicated that the SiO_2_ content of SF is four times higher than the SiO_2_ content of OPC.

### 2.2. Test Methods and Sample Preparation 

The unconfined compression tests (UCT) in this study were carried out according to procedures prescribed by ASTM, with the compression rate of 1 mm/min [33]. A compression machine which can obtain a relatively sufficient amount of data to generate very sensitive stress–strain curves was used for the testing. The induced microstructural changes were also traced using SEM analyses to obtain additional information.

Table 3 summarizes the test programs in this study. For the scenarios listed in Table 3, the mix ratio of cement stabilized soil is characterized by the mass ratio of soil solid (s): dry cement solid (c): water (w). In the tests, the cement content is the mass ratio of cement to the wet soil (the mixture of soil solid and the natural moisture), silica fume content is the mass ratio of SF to cement, water-cement ratio (mass ratio of water to cement and SF) is 0.5.

UCT contains 2 groups. UCT-1 is aimed to investigate the effect of cement content, while UCT-2 is to investigate the effect of SF content. For cement stabilized soil, 5–15% OPC (by dry soil weight) is a typical value, so we set cement content 4–13% (by wet soil weight) [34]. There are generally two mixing methods for silica fume in concrete [35]. One is the internal mixing method, which refers to the substitution of SF for cement. The percentage replacement may vary from 0 to 30 percent, such as 10%, 15% and 20%, and the water-cement ratio remains unchanged [36]; the other one is the external mixing method, whereby SF is mixed into concrete as an external admixture, and the cement content remains unchanged, and the SF content is generally less than 10%, such as 5% [37]. The mechanical properties of the concrete obtained by the external admixture method are higher. Excessive SF is easy to cause the concrete to be too sticky, which is not suitable for construction [38]. It is worth noting that Wong et al. observed that the amount of cement replacement caused strength loss in the early ages (1 to 7 days). Accordingly, in order to study the influence of SF on the strength and microstructure at different ages, after laboratory tests, we have chosen the external mixing method, and the SF content is 0–5% [34]. Zelic et al. found that SF participated in the pozzolanic reaction in 3 days, in order to observe the microstructure of cement stabilized soil in different curing times, we adopted a fixed cement content in UCT-2, the variable is the mass ratio of SF to cement, and the curing time is 3, 7, 14, 28 and 90 days, respectively [39].

In UCT-1, the SF content is 1%, and cement content is 4%, 7%, 10% and 13%, the samples are named SF1C4, SF1C7, SF1C10 and SF1C13, respectively. In UCT-2, cement content is 10%, the SF content is 0%, 0.5%, 1%, 1.5%, 2% and 5%, the samples are named C10SF0, C10SF0.5, C10SF1, C10SF1.5, C10SF2 and C10SF5, respectively.

**Figure 2:**
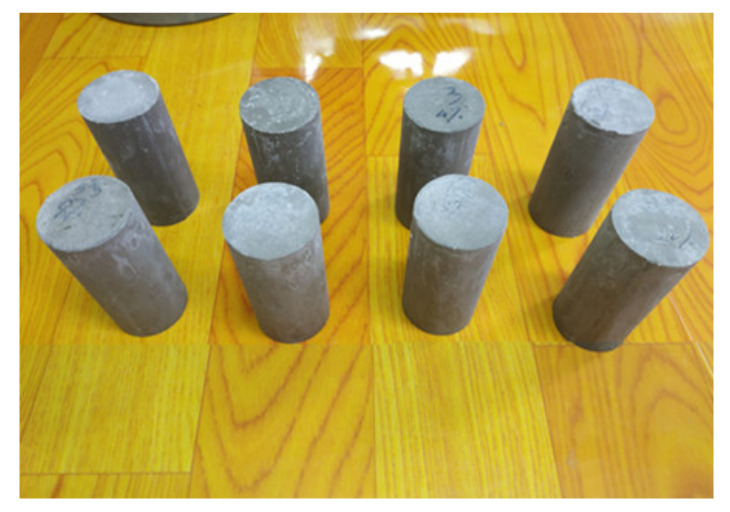
Silica fume cement stabilized soil samples after curing.

In terms of the sample preparation, in order to reduce the impact of impurities, the soft soil was firstly placed in a drying oven at 105 °C for 8 h and crushed, then it was sieved with a 2 mm diameter sieve to remove coarse particles (like shells and small rocks). The soil solid, water, cement and SF are put into the Hobart mixer in proportion to mix thoroughly, then transferred to a polyvinyl chloride mold with a diameter of 39.1 mm and a height of 80 mm, compacted by three layers, each layer was vibrated for 5 min at the shaking table to eliminate the bubbles and ensure its uniformity [40]. After curing for first 24 h, the samples are demolded, then put into deionized water at 20 ± 3 °C for curing. To ensure the accuracy of the tests, there are three parallel samples for each group, the unconfined compression strength (UCS) was determined based on the maximum stress attained or on the stress at 15% axial strain, whichever was obtained first. And the average value of the three parallel samples for each group of data. The unconfined compression tests were performed using a total of 94 samples. Silica fume cement stabilized soil samples after curing are shown in Figure 2, the surface of the sample is flat and smooth.

In addition, the soil samples after curing were studied by SEM for microstructure testing. Take a small piece at the center of the sample section. These pieces were frozen by liquid nitrogen for freeze drying. After that, they were placed in a vacuum machine to sublimate for 24–48 h. After the broken surface was taken to gold plating, it could be used for SEM tests.

## 3. Results

### 3.1. Strength of Cement Stabilized Soil admixed with Silica Fume

**Figure 3:**
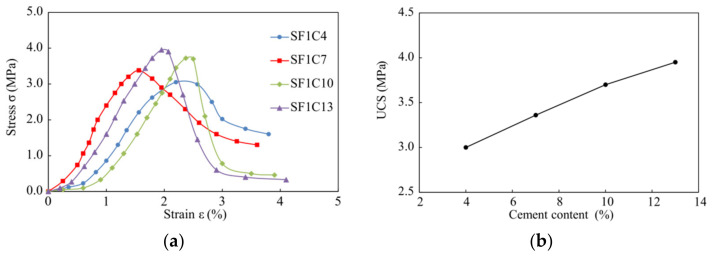
The UCT results for specimens with various cement contents: (**a**) stress–strain curves; (**b**) unconfined compression strength (UCS).

Figure 3a shows the stress–strain curves from UCT-1 for the cement stabilized soil with various cement content. It can be seen that there is an initial slow growth stage for the stress. This is due to the gradual fitting process between the specimen and the apparatus in the initial compression stage. After that, within a certain strain level, the stress increases rapidly with a strain until it reaches the peak (failure stress), and then the stress drops. The failure stress is the unconfined compressive strength (UCS). It can be seen that the UCS value differs due to various cement content. It also exhibits that the post-failure stress of cement stabilized soil with higher cement content (i.e., 10% and 13%) drops much more rapidly than those with lower cement content (i.e., 4% and 7%), which indicates that higher cement content leads to a more brittle material. As illustrated in Figure 3b, the UCS of the cement stabilized soil will increase with cement content. It can be identified that UCS increases almost linearly when cement content grows from 4% to 10%, while its growth rate slows down from cement content of 10% to 13%. So we use a cement content of 10% in the test UCT-2 (see Table 3).

**Figure 4:**
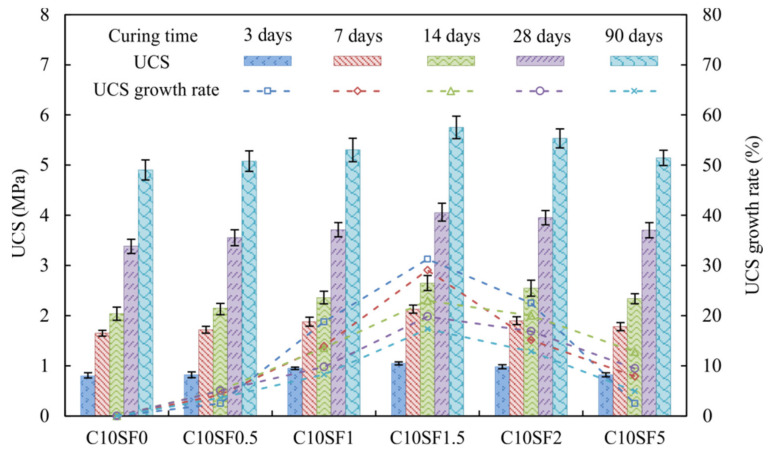
The UCS and UCS growth rate of cement stabilized soft soils with various SF contents.

Unconfined compressive strength (UCS) is one of the most important and basic properties of materials. Figure 4 presents the UCS and UCS growth rate from UCT-2 for cement stabilized soils with various SF contents. The results show that UCS of all cement stabilized soil admixed with SF increased gradually as the curing time increases taking UCS of C10SF0 as reference. However, it does not follow that the higher the content of SF, the higher the value of UCS. When the content of SF is 1.5%, the UCS of cement soil reaches its peak value.

We know that when cement and soft soil are mixed in the presence of water, first the tri-calcium silicate (C_3_S), di-calcium silicate (C_2_S) and tri-calcium aluminate (C_3_A) in the cement are hydrated to form the calcium silicate hydrate gel (CSH), the by-product calcium hydroxide (CH) and Ettringite (Et). Due to the addition of SF, the pozzolanic reaction occurs, and the by-product CH is consumed by the SiO_2_ in the SF and the CSH is formed again, and gradually reacts fully with time. Generally, it includes hydration reaction and Pozzolanic reaction, these reactions are expressed in Equations (1) and (2) below:

OPC hydration reaction:

C_3_S, C_2_S, C_3_A + H_2_O = CSH + CH + Et (1)

Pozzolanic reaction:

CH + SiO_2_ = CSH (2)

Due to the OPC hydration reaction and pozzolanic reaction, the CSH gels formed bond the soil particles together, this binding produces a stronger soil matrix, resulting in an increase in compressive strength. In addition to filling the pores and increasing the bulk density with the addition of SF, the consumption of CH also accelerates the OPC hydration reaction (1). When the CH is consumed and its concentration reduced, the pozzolanic reaction no longer occurs. In addition, the excess SF has no binding properties to the cement stabilized soil under a certain amount of cement. It is adsorbed on the surface of the soil particles due to the van der Waals force, and this molecular force is easily destroyed. Therefore, the influence of SF on the strength of cement stabilized soil has a peak, the optimum SF content is 1.5% as shown in Figure 4. The UCS of C10SF1.5 exceeded 4 MPa after 28 days of standard curing, which was 22% higher than the UCS of C10SF0, the strength improvement is higher than that of bagasse ash, which is also recycled material [41]. As shown in Figure 3b that the UCS of SF1C13 is 3.9 Mpa at 28 days, the UCS of C10SF1.5 is also higher than that of SF1C13, it shows that SF makes the cement stabilized soil strength with 10% cement content higher than that with 13% cement content, and SF can reduce the cement content used and increase the compressive strength significantly. The highest UCS growth rate reached 33% after 3 to 7 days at optimum SF content, indicating that SF particles played a role in the early stage and helped to improve the early-age strength. The specific surface area is 70 times that of cement since the finer particles of SF, the volume of SF is about four times that of cement under the same quality. So SF particles can fill the pores to increase the packing density and accelerate the cement hydration reaction, it has the advantages of great filling effect and good pozzolanic reaction.

### 3.2. Microscopic Characteristics of Cement Stabilized Soil Admixed with Silica Fume Versus Curing Time

The production of CH and CSH in cement stabilized soil directly affects its strength. In order to visually observe the changes of hydration products in cement stabilized soil before and after adding SF, we have observed its microstructure.

**Figure 5:**
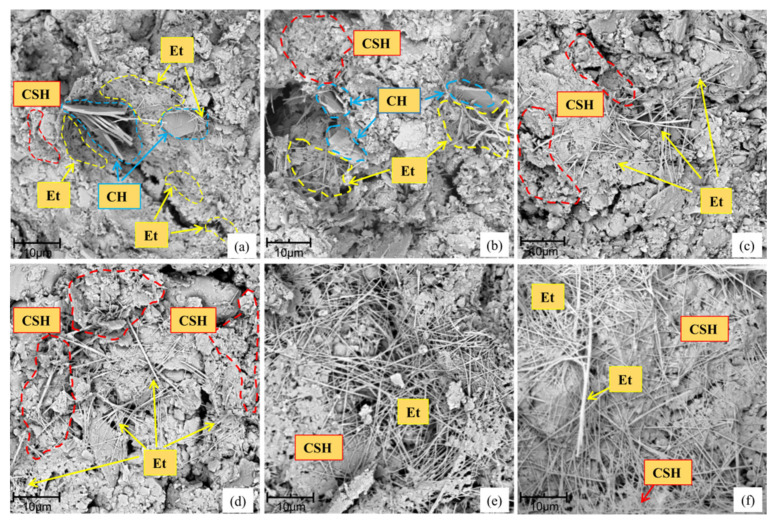
Microscopic images for samples with different curing times: (**a**) C10SF0 at 3 days; (**b**) C10SF1.5 at 3 days; (**c**) C10SF1.5 at 7 days; (**d**) C10SF1.5 at 14 days; (**e**) C10SF1.5 at 28 days; (**f**) C10SF1.5 at 90 days.

Figure 5a is the microscopic image of C10SF0 at 3 days, it shows that porous calcium silicate hydrate gel (CSH), needle-shaped ettringite (Et), and hexagonal plate-shaped calcium hydroxide (CH) crystals are formed after cement hydration reaction, the low strength CH generally exists in the pores, which is the weakest place of cement-based materials. CSH and Et are contributed to the development of strength [42], and the Et crystals are small at 3 days. The SiO_2_ in the SF particles can produce a secondary hydration reaction with CH, which promote the progress of the hydration reaction and generate more CSH. Figure 5b–f shows microscopic images of C10SF1.5 at 3, 7, 14, 28 and 90 days. The edge of CH is incomplete at 3 days, and there is no obvious CH from 7 to 28 days of C10SF1.5, indicating that SF can convert CH to CSH after 3 days, promote the hydration rate and improve the density and help improve early strength of the cement as mentioned earlier. From 3 to 28 days, Et crystals are elongated and thicken gradually, CSH and Et have increased and interwoven into an even framework gradually in the curing time, the soil particles are completely covered by the interweaving of the CSH and needle-like Et after 28 days, and the strength of the porous material is further enhanced. As shown in Figure 5f, the soil particles have been connected by CSH into a whole at 90 days of curing, and the pores have been filled with hydration products, at the same time, the needle-like Et crystals are interwoven into a net, the soil particles are completely covered by the interweaving of CSH and needle-like Et, and the strength of cement stabilized soil is further enhanced. As shown in Figure 4, after 3 to 7 days of curing under the optimal SF content, the highest growth rate of UCS reached 33%, which indicates that the SF played a role in the early stage, helped to accelerate the cement hydration reaction and improve the early-age strength.

### 3.3. Pore Distribution Characteristics of Cement Stabilized Soil with Different Silica Fume Contents

**Figure 6:**
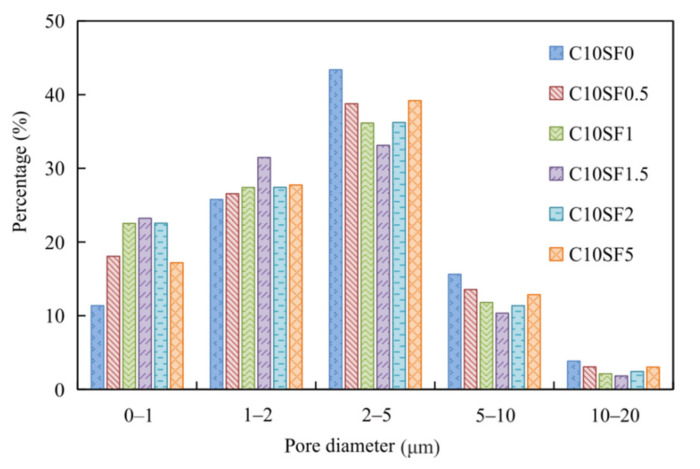
Pore distribution of cement stabilized soil with different SF contents (at 28 days).

In the reinforcement of cement or concrete of engineering, the improvement of material compactness is an important manifestation. From the changes in compressive strength and microstructure in Figure 4 and Figure 5, it can be intuitively seen that silica fume has an effective effect on cement stabilized soil. For the phenomenon whereby excessive silica fume does not increase the strength further, analysis of the pores of the cement stabilized soil and their distribution characteristics is helpful toward the study of the solidification mechanism of the silica fume cement stabilized soil. The pores size and distribution characteristics can be used to explain the changes of compressive strength. We have made statistics on the pore sizes under different silica fume contents through the electron microscope image. Figure 4 shows that the optimum SF content at 28 days is 1.5%. The microscopic images of cement stabilized soil with different SF contents at a magnification of 2000× are selected and IPP (Integrated Performance Primitives) is used to quantitatively analyze the pore diameter. Figure 6 shows the pore diameter distribution with different SF contents at 28 days. It can be seen that all the pore diameter was less than 20 μm, small pores content (0–1 and 1–2 μm) increased and reached the peak when SF content was 1.5% then decreased with the SF content increased, and the medium to large pores content (2–5, 5–10 and 10–20 μm) decreased and reached the minimum when SF content was 1.5% and then increased with the SF content, the maximum increase of pore diameter percentage less than 2 μm was close to 18%. It is consistent with the phenomenon that the strength decreased when the SF content exceeded the optimum. Indicating that SF promotes the hydration reaction of cement and produces more hydration products connect the mineral particles and fill in the pores, so that the pore diameter is reduced and the structure is more dense [22]. When the SF content is too much, CH is consumed and the concentration decreases, making the secondary hydration reaction no longer happen, and the remaining SF particles no longer play a significant role in increasing the strength. SF promotes the hydration reaction of the cement, produces cementitious material CSH and consumes water in the soil at the same time. SF has a large specific surface area, as the amount of cement remains unchanged, with the increase of silica fume content the water demand of the cement stabilized soil also increases. The self-shrinkage increases, so excessive SF will cause micro cracks, which increases the content of macropores and reduces the strength.

## 4. Discussion

Recyclable waste includes domestic waste (eggshell powder), industrial waste (fly ash, silica fume), agricultural waste (bagasse ash) and so on. In soil stabilization, the recyclable waste applications will not only reduce the cost of construction, but can also be of economic importance in both underdeveloped and developing countries. Past studies suggest that silica fume can be successfully used to replace 5–15% of cement in concrete, which offers improvement in the mechanical properties and durability of cementitious materials via the filler effect and pozzolanic reaction [34].

According to the results of this study, it is found that silica fume can be used as an additive to improve soft soil, which improves the efficiency of cement hydration, promotes the production of gel substance CSH and makes the cement stabilized soil more dense. Among industrial wastes, silica fume has been found to be a promising material for soil reinforcement. Silica fume provides more advantages for stabilizing soil. In this study, UCS value of cement stabilized soil with 1.5% SF content reached 4.05 MPa, which is 2.4 times that of fly ash treated soil [43]; compared to the blank sample without SF, the growth rate of cement stabilized soil is 22%, which is higher than that of bagasse ash waste [41]. Another study on black cotton soil carried out by Negi showed that 20% SF content makes the UCS increased by 31% [44]. However, as the result of pore analysis, the extreme fineness of SF increases the water demand, resulting in the generation of micro cracks. Due to the strict requirements of SF on water, the SF amount must be strictly controlled, which limits the improvement of SF to cement stabilized soil. We believe that SF has more room in soft soil improvement. The superplasticizer can be used to make SF play a better role, or silica fume can be used to develop composite additives.

## 5. Conclusions

As an industrial waste, SF was used as an admixture with cement to improve soft soil. The unconfined compressive test (UCT) and the scanning electron microscopy (SEM) test of soil samples at different curing times were used for the cement stabilized soil admixed with SF, and the following conclusions were drawn from the results of this study:

- According to the results of UCT, UCS increases almost linearly within cement content from 4% to 10%, while the growth rate slows down from cement content of 10% to 13%; the SF content has an important influence on the strength characteristics of cement stabilized soil. The UCS of C10SF1.5 at 28 days is 1.22 times that of C10SF0, and is also higher than the UCS of SF1C13, indicating that the addition of SF can reduce the cement content used and increase the compressive strength. The 1.5% SF of the cement content is considered optimum, excessive SF will not further increase the strength. SF played a role in the early stage, the UCS growth rate of 3 days exceeded 33%, it helped to accelerate the cement hydration reaction and significantly improve the early-age strength of stabilized soil, which can improve construction efficiency in engineering.
- SEM analysis shows that SF started to react with calcium hydroxide (CH) at 3 days, which prevents CH from forming weak zones in the pores and promotes cement hydration to form calcium silicate hydrate gel (CSH), the formed CSH bonds the soil particles together gradually, this binding produces a stronger soil matrix with the increase of curing time, resulting in an increase in compressive strength.
- According to the pores size analysis of cement stabilized soil with different SF content in the standard age of 28 days, when the SF content is lower than the optimum, the small pores of the cement stabilized soil increase due to the generation of CSH, and the optimum SF content helps the cement stabilized soil to be more uniform. The increase in water demand with excessive SF will increase the self-shrinkage of cement stabilized soil and produce micro cracks, which are manifested by the increase of large pores and the decrease of compressive strength of cement stabilized soil.

Silica fume can be used to improve the soft soil of civil engineering to reduce environmental pollution problems, which could increase the value of industrial waste. Therefore, the use of silica fume is very useful in sustainable building technology.

## Figures and Tables

**Table 1:** Physical properties of soft soil.

Specific Gravity	Natural Moisture Content (%)	Wet Density (g/cm^3^)	Initial Void Ratio	Liquid Limit (%)	Plasticity Index (%)	Sand (%)	Silt (%)	Clay (%)
2.68	45.0	1.75	1.21	41.8	20.3	1.8	68.1	30.1

**Table 2:** Chemical compositions of soft soil, silica fume (SF) and ordinary Portland cement (OPC).

Components	SiO_2_	Al_2_O_3_	Fe_2_O_3_	MgO	CaO	Na_2_O	K_2_O	Other
SF (%)	95.02	0.43	0.08	0.64	0.45	0.34	1.12	1.89
Soft soil (%)	60.56	18.24	8.09	2.86	1.95	1.47	3.48	3.32
OPC (%)	21.75	5.66	4.63	1.69	64.29	0.41	0.76	0.81

**Table 3:** The unconfined compressive test (UCT) programs.

Number	Mix Ratio (s:c:w)	SF Content (%)	Cement Content (%)	Symbol	Curing Time (Day)
UCT-1	100:5.8:47.93	1	4	SF1C4	28
	100:10.2:50.13		7	SF1C7	
	100:14.5:52.32		10	SF1C10	
	100:18.9:54.52		13	SF1C13	
UCT-2	100:14.5:52.25	0	10	C10SF0	3,7,14,28,90
	100:14.5:52.29	0.5		C10SF0.5	
	100:14.5:52.32	1		C10SF1	
	100:14.5:52.36	1.5		C10SF1.5	
	100:14.5:52.40	2		C10SF2	
	100:14.5:52.61	5		C10SF5	

## Data Availability

Data is contained within the article.
